# Typhoid Gangrene of the Lower Extremities

**Published:** 1904-01

**Authors:** B. Merrill Ricketts

**Affiliations:** Cincinnati, Ohio.


### BUFFALO MEDICAL JOURNAL.

VOL. XLIII.—LIX. JANUARY, 1904. No. 6.

### ORIGINAL COMMUNICATIONS.

### Typhoid Gangrene of the Lower Extremities.

**One Hundred and Thirty-four Cases—Spontaneous and Surgical Amputations—A Historical Resume.**[1]

By B. MERRILL RICKETTS, PH. B., M. D., Cincinnati, Ohio.

COMPARATIVELY few cases of gangrene of any locality are associated with typhoid. The disease occasionally attacks the lips, tongue, cheeks and genitalia. It has also been observed involving the fingers, hands, arms, toes, feet, and legs. It is gangrene of the feet and legs which will be considered at this time.

Gangrene of the lower extremities associated with typhoid fever is indeed rare compared with the great number of cases of typhoid fever throughout the world. There is no known cause; climate, habits, occupation and general environment do not offer any solution of the problem. It appears to be due to the bacillus typhosus, but this has not been proven. That the subject may be more fully considered the brief statistics of the reported cases are appended. Doubtless many more cases have occurred than those herein mentioned.

*Pathology.*—Gangrene of any part associated with typhoid is due to embolism, thrombosis, or inflammation of the veins or arteries, one or both, but more frequently the arteries. Gangrene is due to a plug, inflammation, anomalies and obstruction to collateral circulation and may appear at the crisis of disease, or at any time during the progress of the fever or during convalescence from the same.

When due to inflammation the process, as a rule, extends until all of the vessels of the extremities are involved, while in cases

1. Read before the Virginia State Medical Society, at Roanoke, September, 1903.

of embolism or thrombosis the process is more localised, with partial or complete destruction of the tissues beyond the occlusion, which may be at any point in the course of the vessel. The gangrene may involve a part or all of the circumference of the leg, usually a part when there is thrombophlebitis.

When both veins and arteries are involved, or arteries alone, the entire circumference is affected; there may or may not be tenderness along the course of the vessels. This is more frequently so if the veins be involved. The gangrene may be confined to the course of the artery involved with severe hyperesthesia. Occlusion of vessel or vessels may be partial or complete. One or both external iliac arteries or veins or all may be embolic, partial or complete, and partial paralysis may follow without any atrophy. Gangrene of feet and legs when double may be simultaneous or there may be an interval of days or weeks.

Fever may be high or low, or there may be none at all. Gangrene may be moist or dry, slow or rapid in its progress. Metastatic abscess may form in the kidney, liver, lung, spleen, pancreas, heart, or brain, and there may be spontaneous fracture of the long bones.

*Surgical Amputation.*—Amputation should be made as soon as gangrene is discovered and far enough above the diseased tissues to be reasonably sure that nothing but healthy structures and especially healthy bloodvessels are divided. If the lower leg is involved amputation should be above the knee-joint (lower third of thigh), as in such cases the occlusion is almost invariably in the popliteal artery. Mortality in both spontaneous and surgical amputation is higher in subjects over 40 years of age, and when amputation is delayed.

If there is great debility and surgical anesthesia contraindicated (which is seldom found), a rapid primary amputation should be made through the gangrenous tissues. The secondary amputation can then be made subsequently so that the work may be completed in two sittings. There are probably but few cases in which this course is necessary, or is to be advised. There are a few cases in which the gangrene will continue even after the amputation is made in normal tissue.

The more favorable cases for amputation are those involving the toes, feet and lower leg during convalescence. This is so in amputation of the lower extremities for any purpose, the mortality increasing as the upper thigh is approached.

Spontaneous amputation, as a rule, should not be encouraged. However, in a few cases in the aged where both extremities are involved with dry gangrene, slow in its development, with great debility of any kind of surgical anesthesia being contraindicated, it should be carefully considered. If, however, the line of demarcation is established and amputation can be done rapidly above the knee or ankle without surgical anesthesia then spontaneous amputation should not be considered.

*Personal Experience.* CASE.—I was called in consultation with Dr. Loomis, Independence, Ky., in January, 1902, to see a married woman, aged 28 years, who had borne 2 or 3 children, and who was about the thirtieth day in typhoid fever. The right leg was gangrenous to the middle third of thigh, moist gangrene having first appeared in the toes eight or ten days previous to my visit. Temperature not unusual and with the exception of an endocarditis she was rather comfortable.

Amputation at the upper third of the thigh was decided upon and done immediately under the influence of chloroform. Time of amputation 1½ minutes. The femoral vessels were occluded at the point of the first incision, but the femoral artery was opened with the snip of the scissors and ligated. The soft tissues were sutured with silkworm gut and drainage provided for with gauze. The shock was but slight and of little consequence. She rallied promptly, and did very well for 7 or 8 days, when she began to decline and dissolution took place on the tenth day.

Such cases are rare, and it is reported because of its rarity; also, that it may be considered with other amputations of the lower extremities for gangrene resulting from occlusion of the vessels during the progress of typhoid infection. This is one of three recorded case where amputation was made at the upper third of the thigh, and while death ensued 10 days after, the lesson to be taught is that such an amputation can of itself be made under such circumstances without fatality. For, if death had resulted from the operation in this case, it would have occurred within a few hours. The gangrene continued to extend after the operation and her death was, no doubt, due to the infection resulting from gangrene.

In two cases recorded in which recovery took place amputation was made of one leg and one thigh in each case. In two other cases both legs were amputated with recovery. It is reasonable to presume that the same rule holds good as to the amputation of the thigh for typhoid gangrene as it does for other causes. The question is first, should surgical amputation be resorted to in cases of gangrene of the lower extremities during typhoid fever, and should not the line of demarcation be allowed to form before surgical measures are adopted, or, at least, should it not be delayed until the patient's physical condition improves?

*Statistics.*—Of 134 reported cases 100 were male, 34 females; 128 surgical and 3 spontaneous amputations, 3 not stated; 15 both legs, 41 right leg, 20 left leg, 15 both feet, 18 right foot, 16 left foot, 5 toes, 1 hands and toes, 3 middle thigh, 3 upper thigh, 2 lower thigh, 3 both thighs; 20 dry and 2 moist gangrene, 112 not stated; 6 recoveries with operation, 1 no operation; 34 deaths without operation, 22 deaths with operation, 12 unknown.

408 BROADWAY.

